# Social vulnerability influences racial and ethnic disparities in *Clostridioides difficile* infection outcomes

**DOI:** 10.1017/ice.2025.57

**Published:** 2025-06

**Authors:** Jacinda C. Abdul-Mutakabbir, Karen K. Tan

**Affiliations:** 1 Division of Clinical Pharmacy, Skaggs School of Pharmacy, University of California San Diego, La Jolla, CA, USA; 2 Division of the Black Diaspora and African American Studies, University of California San Diego, La Jolla, CA, USA; 3 Department of Pharmacy, Loma Linda University Medical Center, Loma Linda, CA, USA

To the Editor- We previously reported racial and ethnic differences in the clinical outcomes of patients diagnosed with *Clostridioides difficile* infections (CDI) at Loma Linda University Medical Center (LLUMC). Our findings indicated that racially and ethnically minoritized (REM) individuals were more likely to be diagnosed with a CDI compared to non-Hispanic White or non-REM (n-REM) individuals.^1^ Furthermore, minoritized individuals were more likely to present with a fulminant CDI compared to their n-REM counterparts. While our study aligns the existing literature on racial inequities in the incidence of CDI and severity of infection, it did not fully explain the underlying causes of these observed differences.^2,3^

It is important to recognize that race is a social construct rather than a biological determinant of health. However, the experiences of systemic racism and discrimination have significant societal consequences, particularly related to social determinants of health (SDoH). Disparities across a continuum of SDoH—including education, socioeconomic status, environment, and access to healthcare—have been shown to contribute to inequitable outcomes in infectious diseases among REM groups.^4,5^ This led us to revisit our previously published data to explore whether SDoH contributed to the reported racial disparities in CDI outcomes and identify actionable areas for interventional change.

The Centers for Disease Control and Prevention’s (CDC) Social Vulnerability Index (SVI) provides a composite measure of neighborhoods (census tracts) based on four major subthemes: socioeconomic status (SES), housing characteristics (H&C), race, ethnicity, and language (REM) status, and housing and transportation (H&T).^6^ The SVI composite score has been used as a surrogate to describe SDoH inequities in CDI outcomes.^5^ However, the SVI score has not yet been deconstructed by subthemes to describe differences in the social vulnerability scoring among patients diagnosed with CDI—varying in clinical severity.

To address this gap, we geocoded the addresses of the 219 patients from our original data set (adult patients with an initial CDI case admitted between January 2020 to June 2021) and mapped them using the CDC SVI tool. We calculated both the overall SVI composite score and subtheme scores for individuals diagnosed with CDI (non-severe, severe, and fulminant). For ease of comparison, the individuals were assigned into two main groups: patients with SVI scores of <0.4999 (indicating a low to low-medium scoring) were allocated to the low vulnerability (LV) scoring group, and patients with SVI scores of ≥ 0.5 (indicating a medium-high to high scoring) were allocated to the high vulnerability (HV) scoring group.

After excluding 13 patients without an identifiable census tract, 206 remained in the final analysis. Overall, a total of 88 patients (43%) were diagnosed with non-severe CDI, 80 patients (39%) with severe CDI, and 38 patients (18%) with fulminant CDI. Among those with non-severe CDI, 70/88 patients (80%) had an HV overall composite score. For the subtheme scoring of these patients, 74% had an HV score in SES, 65% had an HV score in H&C, 92% had an HV score for REM status, and 59% had an HV score in H&T. Among those with severe CDI, the majority of patients 65/80 (81%), had an HV overall composite score. When considering the patients’ subtheme scoring, 76% had an HV score in SES and H&C, 94% had an HV score for REM status, and 68% had an HV score in H&T. Similarly, among the 38 patients with fulminant SVI, the majority of patients 30/38 (79%) had an HV overall composite score. Within this group, 71% of the patients had an HV score for SES and H&C, 97% had an HV score for REM status, and 76% had an HV score in H&T. The breakdown of the CDC SVI overall composite and subtheme scoring, including counts and percentages, are shown in Table 1.

**Table 1.** CDC SVI composite and subtheme scoring for patients with severe and fulminant CDI^a^

Vulnerability group	Non-severe CDI	Severe CDI	Fulminant CDI
Overall SVI			
Low vulnerability	18/88 (20%)	15/80 (19%)	8/38 (21%)
High vulnerability	70/88 (80%)	65/80 (81%)	30/38 (79%)
Socioeconomic status			
High vulnerability	65/88 (74%)	61/80 (76%)	27/38 (71%)
Household characteristics			
Low vulnerability	31/88 (35%)	19/80 (24 %)	11/38 (29%)
High vulnerability	57/88 (65%)	61/80 (76%)	27/38 (71%)
Race and ethnic minority status			
Low vulnerability	7/88 (8%)	5/80 (6%)	1/38 (3%)
High vulnerability	81/88 (92%)	75/80 (94%)	37/38 (97%)
Housing type and transportation			
Low vulnerability	36/88 (41%)	26/80 (33%)	9/38 (24%)
High vulnerability	52/88 (59%)	54/80 (67%)	29/38 (76%)

a Shown in this table are the CDC SVI overall composite score and four subthemes used to describe the influence of SDoH factors on CDI (non-severe, severe, and fulmimant presentations). The breakdown of these themes are as follows: Socioeconomic Status (poverty level, unemployment, housing cost burden, education, and insurance coverage); Household Characteristics (age, disability status, single-parent households, and English proficiency); Racial & Ethnic Minority Status (Hispanic/Latino, Black, American Indian, Pacific Islander, and multiracial populations); and Housing Type & Transportation (multi-unit housing, mobile homes, overcrowding, lack of vehicle access, and group quarters).

These findings reinforce the link between social vulnerability and CDI, including non-severe, severe, and fulminant presentations of the disease. As expected, most individuals diagnosed with CDI had an HV score in the REM subtheme, reflecting the racial disparities observed in our initial study. Nonetheless, the high proportion of HV scoring for the SES, H&T, and H&C subthemes suggests that the broader socioeconomic and environmental factors contribute to deleterious CDI outcomes. Moreover, these vulnerabilities may create barriers to managing chronic conditions such as chronic kidney disease (CKD), which is known to increase CDI susceptibility, severity, and related mortality.^3,7^ Notably, in our initial study, a pre-existing CKD diagnosis was shown to partially mediate the relationship between race/ethnicity and severe and fulminant CDI.^1^

Our findings also led us to explore potential institutional changes in CDI management through collaboration with the LLUMC Healthcare Equity Committee. We conducted a retrospective review of the initial cohort of 219 patients hospitalized with CDI, examining their encounters within the 30 days leading up to their hospitalization. Our focus was on emergency department (ED) and primary care visits related to gastrointestinal (GI) or abdominal symptoms to identify missed opportunities for early detection.

In our review, we found that 11 patients had a visit with LLUMC 30 days before their hospitalization, where they presented with GI symptoms potentially associated with CDI. Most of the encounters were non-emergent telephone appointments. Of these 11 patients, seven (64%) identified as REM, and six out of 7 patients (86%) had an overall composite HV score, and HV scores in each subtheme. Notably, five of the six highly vulnerable REM individuals (83%) were diagnosed with severe CDI, and one individual (17%) was diagnosed with fulminant CDI. This highlights a potential opportunity for improved screening for early CDI identification.

Therefore, we concluded that improving screening practices during remote consultations by incorporating targeted diagnostic questions—especially for REM individuals who live in highly vulnerable communities—could enhance early detection of CDI. Ultimately, this early detection may help prevent severe or fulminant disease presentations. However, due to the small sample size, further investigation is needed to assess the impact of such interventions on patient outcomes.

The CDC SVI is an important tool for measuring social risk factors. When incorporated into research that assesses clinical outcomes, it can provide insights into the economic and environmental factors contributing to disparities in healthcare-associated infections among different racial and ethnic minoritized groups. Previous studies have indicated that a high composite vulnerability score, or elevated scores in the socioeconomic subtheme, are positively correlated with racial and ethnic inequities.^8,9^ Our analysis suggests that a more detailed approach—calculating scores for each subtheme rather than relying solely on composite scores or just the socioeconomic subtheme—may reveal additional insights into health disparities. We encourage future researchers to thoroughly examine the impact of social factors on infectious disease outcomes and to develop effective interventions to address these inequities.

## References

[1] Lee JM , Zhou AY , Ortiz-Gratacos NM , et al. Examining the impact of racial disparities on Clostridioides difficile infection outcomes at a Southern California academic teaching hospital. Infect Control Hosp Epidemiol 2023;44:1–5. 10.1017/ice.2023.84 PMC10665859 37138348

[2] Yang S , Rider BB , Baehr A , Ducoffe AR , Hu DJ. Racial and ethnic disparities in health care–associated Clostridium difficile infections in the United States: state of the science. Am J Infect Control 2016;44:91–96. 26454749 10.1016/j.ajic.2015.08.007

[3] Mao EJ , Kelly CR , Machan JT. Racial differences in Clostridium difficile infection rates are attributable to disparities in health care access. Antimicrob Agents Chemother 2015;59:6283–6287. 26248363 10.1128/AAC.00795-15 PMC4576108

[4] Marcelin JR , Hicks LA , Evans CD , et al. Advancing health equity through action in antimicrobial stewardship and healthcare epidemiology. Infect Control Hosp Epidemiol 2024;45:412–419. 38351853 10.1017/ice.2024.7 PMC11318565

[5] Butler JL , Hranac R , Johnston H , et al. Association of Clostridioides difficile infection rates with social determinants of health in Denver area census tracts, 2016–2019. Prev Med Rep 2023;36:102427. 37766722 10.1016/j.pmedr.2023.102427 PMC10520868

[6] Centers for Disease Control and Prevention. ASTDR CDC Social Vulnerability Index. https://web.archive.org/web/20250121103056/https://atsdr.cdc.gov/place-health/php/svi/svi-interactive-map.html. Published 2024. Accessed February 2025.

[7] Reveles KR , Strey KA , Abdul-Mutakabbir JC , Mendoza VM , Carreno JJ. Infectious inequity: how the gut microbiome and social determinants of health may contribute to Clostridioides difficile infection among racial and ethnic minorities. Clin Infect Dis 2023;77:S455–S462. 38051968 10.1093/cid/ciad586 PMC10697666

[8] Rha B , See I , Dunham L , et al. Vital signs: health disparities in hemodialysis-associated staphylococcus aureus bloodstream infections - United States, 2017–2020. MMWR Morb Mortal Wkly Rep 2023;72:153–159. 36757874 10.15585/mmwr.mm7206e1 PMC9925139

[9] Brown DR , Henderson HI , Ruegsegger L , Moody J , van Duin D. Socioeconomic disparities in the prevalence of multidrug resistance in Enterobacterales. Infect Control Hosp Epidemiol 2023;44:2068–2070. 37385945 10.1017/ice.2023.116 PMC11975411

